# Correlation of multifidus degeneration with sex, age and side of herniation in patients with lumbar disc herniation

**DOI:** 10.1186/s12891-023-06783-2

**Published:** 2023-08-16

**Authors:** Guogang Tian, Yi Wang, Jiao Xia, Jiang Wen, Tao Li, Yuewen Li, Guogang Dai

**Affiliations:** Cervicodynia/Omalgia/Lumbago/Sciatica Department 2, Sichuan Provincial Orthopedic Hospital, 132 West First Section First Ring Road, Chengdu, Sichuan Province China

**Keywords:** Lumbar disc herniation, Multifidus degeneration, Age, Sex, Herniated side

## Abstract

**Objective:**

The aim of this study was to determine the relationship between multifidus degeneration and sex, age and side of protrusion in patients with lumbar disc herniation(LDH).

**Methods:**

Data were collected from September 2015 to September 2022 from patients with L4/5 and L5/S1 LDH. A total of 104 patients (62 males and 42 females) were included in this study, and there were 35 and 69 cases of L4/5 and L5/S1 LDH, respectively. Patients were divided into 4 groups according to age: group 1 (20–29), group 2 (30–39), group 3 (40–49) and group 4 (50–59). Magnetic resonance spectroscopy analysis was used to observe the fat fraction (FF) and functional cross-sectional area (f-CSA) of the defatted multifidus muscle of the protruding side (affected side) and the nonprotruding side (healthy side) of the L4/5 and L5/S1 gaps to evaluate the relationship between multifidus degeneration and sex, age and protruding side in patients with LDH.

**Results:**

Between sexes, the FF of the multifidus muscle was significantly greater in women than in men, regardless of whether it was on the affected or healthy side of the L4/5 segment or on the affected or healthy side of the L5/S1 segment (P < 0.05). Between age groups, there was a significantly positive relationship between the change in FF (%) of the multifidus muscle in patients with LDH and age, with increasing fatty infiltration of the multifidus increasing with age (P < 0.05); notably, there was a significant difference between group 4 and the remaining three groups but no significant difference between groups 1, 2 and 3. The f-CSA of the multifidus (cm^2^) was negatively correlated with age, with the f-CSA of the multifidus becoming more atrophic with increasing age; specifically, there was a significant difference between group 1 and the other three groups (P < 0.05) but not between groups 2, 3 and 4. Regarding the side of the herniated disc, (1) the differences in FF and f-CSA at the L4/5 and L5/S1 levels were not statistically significant between the affected side and the healthy side in patients with lumbar disc herniation at the L4/5 segment (P > 0.05); (2) the differences in FF and f-CSA at the L5/S1 level were not statistically significant between the affected side and the healthy side in patients with LDH at the L5/S1 segment (P > 0.05); (3) the difference between FF at the L4/5 level and f-CSA and FF at the L5/S1 level was not statistically significant (P > 0.05); and (4) the f-CSA at the L5/S1 level was significantly greater on the healthy side than on the affected side (P < 0.05).

**Conclusion:**

The proportion of lipoatrophy in female patients with L4/5 and L5/S1 disc herniations was greater than that in male patients. Lipoatrophy of the multifidus muscle increased with age and was significantly worse in patients over 50 years of age. The f-CSA of the multifidus muscle was negatively related to age, and the f-CSA of the multifidus muscle became more atrophic with increasing age. A comparison of degeneration showed no significant difference between the L4/5 patients and the L5/S1 patients in terms of f-CSA atrophy on the affected side of the herniated disc compared to the healthy side.

## Background

Lumbar disc herniation (LDH) is a common orthopaedic condition with a population prevalence of up to 40% that occurs due to degeneration and herniation of the intervertebral disc and a series of corresponding symptoms and signs caused by biochemical stimulation of the disc tissue and mechanical compression of the nerve roots [1, 2]. The many factors involved in lumbar disc herniation have been comprehensively analysed, of which lumbar stability and lumbar degeneration have been a focus. Among the many factors that maintain the stability of the lumbar spine, the paravertebral muscles are important structures. The multifidus muscle, located at the most medial aspect of the spine, is the paravertebral muscle with the largest attachment area, and it controls spinal motion superficially and deep intersegmental motion. The multifidus muscle is located above and below the intervertebral discs, immediately above and below the vertebral body and disc. This muscle acts as a viscoelastic material, like a rubber fixture applied to the long axis of the vertebral body, which serves to distribute the forces on the disc when stresses are generated in the vertebral body disc. The multifidus muscle does not focus on the direct movement of the lumbar vertebrae but rather on the contraction of the muscle. When the spine is out of balance, the multifidus muscle is the first to contract, increasing tension in the lumbar segments and reducing undesirable displacement and rotation of the spinal segments. The multifidus muscle is therefore closely related to spinal stability. When the multifidus muscle becomes strained, torn or even calcified, the corresponding vertebral body becomes locally unstable and this spinal instability indirectly affects the stresses on the disc and vertebral body of the segment. MRI was found to be important for quantitative scoring of lumbar spine muscle morphology and function by Zhang M et al. [3]. His study found that multifidus degeneration was more pronounced in patients with lumbar disc herniation than in healthy individuals. Ozcan-Eksi EE et al. [4]. used MRI to examine the degeneration of lumbar muscles and discs in patients with lumbar disc herniation by sex and found a close relationship between the two variables. There were also differences in fatty infiltration of the multifidus muscle between the different sexes. Similarly, Paalanne N et al. [5]. found that there was asymmetry in the degeneration of the lumbar multifidus muscle in patients with unilateral herniated discs, indicating that the degree of degeneration and fatty infiltration of the affected side of the multifidus muscle was significantly greater than that of the healthy side, and that there was a strong correlation between the herniated side and the degeneration of the multifidus muscle. In this study, magnetic resonance spectroscopy was used as a technical tool to compare the relationship between sex, age and side of the herniated disc and multifidus degeneration in patients with lumbar disc herniation and to provide a reference for the clinical diagnosis and treatment of lumbar disc herniation.

## Materials and methods

### General information

One hundred and four patients with L4/5 and L5/S1 lumbar disc herniation admitted to Sichuan Orthopaedic Hospital from September 2015 to September 2020 were selected for the study, namely, 62 males (59.6%) and 42 females (40.4%); among them, the oldest was 59 years old, and the youngest was 20 years old, with an average age of 41.43 ± 10.03 years. The study included 35 (33.7%) and 69 (66.3%) cases of L4/5 and L5/S1 lumbar disc herniation, respectively. The study was reviewed and approved by the ethics committee of our hospital, and all patients signed the informed consent form.

### Inclusion and exclusion criteria

The case inclusion criteria were as follows: (1) age of 20–59 years; (2) BMI 18–25 kg/m2; (3) diagnosed with lumbar disc herniation according to the North American Spine Society (NASS) recommended treatment guidelines [6]; and (4) categorized by scholars at Michgan State University (MSU), USA.Herniated discs in zones AB, B, C and BC with unilateral symptoms were included in the test group. Patients voluntarily enrolled in the study and signed the informed consent form.

The exclusion criteria were as follows: (1) tumour, osteoporosis, trauma, slippage, stenosis, infection, scoliosis and other deformities; (2) neuromuscular, metabolic and rheumatic immune diseases; (3) protrusion of more than one segment; and (4) history of previous spinal surgery. Patients with other conditions deemed inappropriate by the investigator for study participation were also excluded.

### Radiographic measurements

**Fig. 1:**
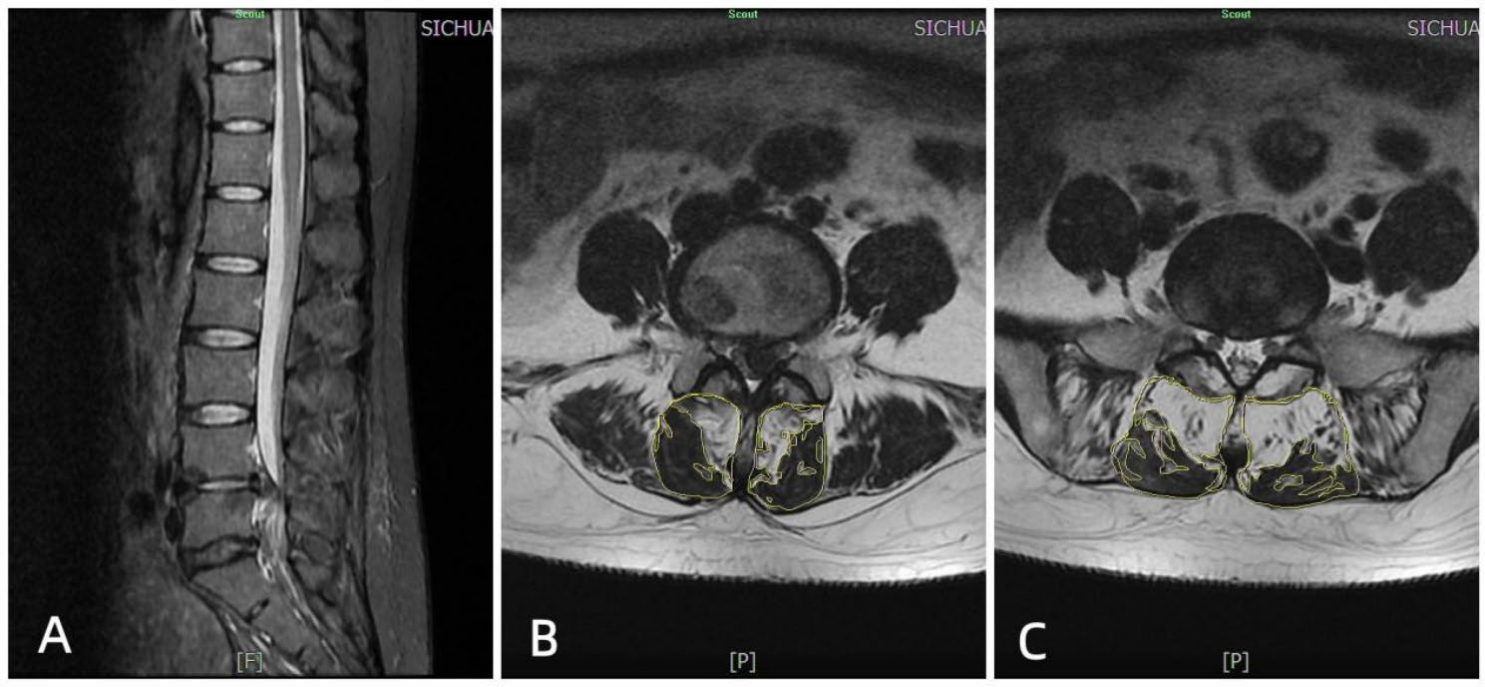
Shows random a L4/5 disc herniation. Figure A shows sagittal MRI. Figure B shows the percentage of fat in the multifidus muscle at the L4/5 level in this patient (39.4% on the affected side and 36.8% on the healthy side), and Figure C shows the percentage of fat in the multifidus muscle at the L5/S1 level in this patient (49.4% on the affected side and 42.6% on the healthy side)

In this study, a conventional T2WI serial horizontal position scan of the lumbar spine was performed using a Siemens superconducting MRI above 1.5 T at Sichuan Orthopaedic Hospital. All patients were evaluated by senior radiographers, with the centre point of the lesioned segment (L4/5 or L5/S1) as the measurement level, and images were acquired using ImageJ processing software (Fig. 1) along the outer contour of the multifidus muscle, thereby avoiding the fatty tissue around the muscle. The cross-sectional area (CSA) of the multifidus muscle on the target image was selected, and then the intramuscular fat was further selected; the system automatically generated the fat fraction (FF) and functional CSA (f-CSA)(defatted CSA of the multifidus muscle) of the drawn area, which was averaged three times for each of the left and right sides. The CSA was divided into total CSA (total muscle area within the fascia, including adipose tissue), functional CSA (f-CSA) (defatted CSA of the multifidus) and fat CSA (fatty area within the muscle). The fat fraction (FF) = fat CSA/total CSA and was averaged between two orthopaedic surgeons with at least 5 years of experience.

### Statistical processing

SPSS 26.0 software was used for statistical analysis of the measured data. Count data are expressed as the number of cases and percentages. For measurement data, the Shapiro‒Wilk test was first used to test for normal distribution and the chi-square value; if normality was satisfied, the mean ± standard deviation ($\bar x$±s) was used for statistical description, and the t test and one-way ANOVA were used for comparison between two and more groups; otherwise, the median (interquartile spacing) was used for statistical description, and the nonparametric rank sum test was used to compare groups. Spearman’s correlation analysis was used to assess the correlation between the rate of multifidus lipofuscinosis and age. All of the above analyses were considered statistically significant at P < 0.05.

## Results

### Comparison of basic patient information

**Fig. 2:**
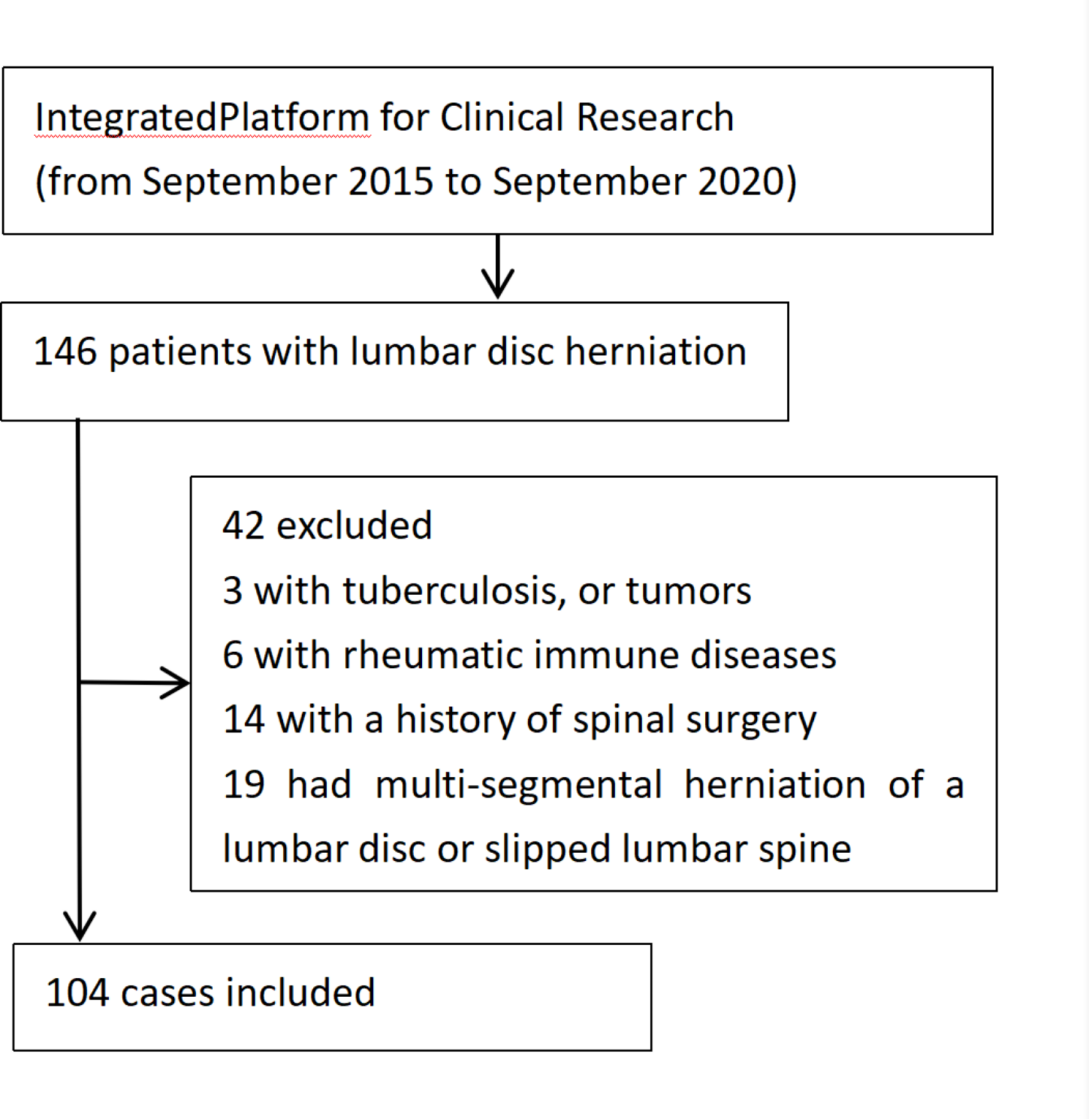
Flow chart

A total of 104 eligible patients with lumbar disc herniation were included in this study, namely, 62 males and 42 females. Patient age ranged from 20 to 59 years old, with an average of 41.3 years old. Eighteen patients were in group 1 (20–29 years old), 21 patients were in group 2 (30–39 years old), 45 patients were in group 3 (40–49 years old) and 20 patients were in group 4 (50–59 years old). Thirty-five patients had L4/5 herniation, and 69 patients had L5/S1 herniation (Table 1).The flow chart is shown below. (Fig. 2)

**Table 1:** General Information

Sex		Age ($\bar x$±s) (years) 41.43 ± 10.03				Prominent segments	
Male No. (%)	Female No. (%)	(20–29) N**o.**	(30–39) N**o.**	(40–49) N**o.**	(50–59) N**o.**	L4/5 No. (%)	L5/S1 No. (%)
62(59.6)	42 (40.4)	18	21	45	20	35 (33.7)	69 (66.3)

### Comparison of the percentage of fat in the multifidus muscle by sex

Since men and women have significant differences in body composition and muscle dimensions, only the fat percentage of the multifidus muscle was compared. The percentage of fat in the multifidus muscle was significantly greater in females than in males, regardless of whether it was on the affected or healthy side of the L4-5 segment or on the affected or healthy side of the L5-S1 segment (P < 0.05) (Table 2).

**Table 2:** Comparison of multifidus fat percentage by sex

Classification	Male	Female	t value	P value
L4/5 level (i)FF(%)	18.09 ± 6.49	22.83 (12.88)	-4.449	0.000
L4/5 level (ii)FF(%)	19.44 ± 6.44	22.07 (9.66)	-2.983	0.004
L5/S1 level (i)FF(%)	23.18 (9.95)	28.48 ± 9.57	-2.265	0.026
L5/S1 level (ii)FF(%)	23.09 ± 7.99	26.95 ± 7.12	-2.520	0.013

Notes:(i) means: the affected side ;(ii) means: the healthy side

### Relationship between different age groups and the lipid ratio of the multifidus muscle in patients with lumbar disc herniation

#### Correlation between age and Spearman’s ratio of multifidus degeneration in patients with lumbar disc herniation

There was a positive relationship between lumbar disc herniation and age, with increasing fatty infiltration of the multifidus muscle with increasing age. The f-CSA of the multifidus muscle (cm^2^) was negatively correlated with age, with the f-CSA of the multifidus muscle becoming more atrophic with increasing age (Table 3).

**Table 3:** Correlation between age and degeneration of the multifidus muscle in patients with lumbar disc herniation

	Multifidus change in FF (%)				Multifidus functional CSA (cm^2^)			
	L4/5(i)	L4/5(ii)	L5/S1(i)	L5/S1(ii)	L4/5(i)	L4/5(ii)	L5/S1(i)	L5/S1(ii)
Year	0.371	0.245	0.347	0.280	-0.296	-0.337	-0.273	-0.259
Age	0.000	0.012	0.000	0.004	0.002	0.000	0.005	0.008

Notes: (i) means: the affected side; (ii) means: the healthy side

#### Comparison of multifidus degeneration in patients of different ages with mid-lumbar disc herniation

FF on the affected side at the L4/5 level and FF on the affected and healthy sides at the L5/S1 level showed that the degree of multifidus lipofuscinosis was greater in group 4 than in groups 1, 2 and 3; additionally, there was no significant difference between groups 1, 2 and 3. FF on the healthy side at the L4/5 level showed that the degree of multifidus lipofuscinosis was greater in group 4 than in groups 2 and 3. Nevertheless, there was no significant difference between groups 1, 2 and 3; no significant difference between group 1 and groups 2, 3 and 4; and no significant difference between groups 2 and 3.

Based on the affected and healthy sides at both the L4/5 and L5/S1 levels, the functional CSA (f-CSA) of the multifidus was significantly greater in group 1 than in the remaining three groups; additionally, there was no significant difference between groups 2, 3 and 4 (Table 4).

**Table 4:** Differences in multifidus degeneration in patients with mid-lumbar disc herniation by age group (M ± SD)

	Group1(n = 18)	Group2(n = 21)	Group3 (n = 45)	group4(n = 20)	F	LSD
L4/5 level (i)FF(%)	17.65 ± 7.86	19.25 ± 7.51	20.27 ± 6.73	26.37 ± 8.44	5.207**	1,2,3 < 4
L4/5 level (ii)FF(%)	21.85 ± 5.72	18.47 ± 6.41	19.8 ± 5.57	25.78 ± 8.85	5.271**	2,3 < 4
L5/S1 level (i)FF(%)	23.52 ± 7.47	24.00 ± 7.84	25.15 ± 7.64	33.18 ± 9.67	6.408**	1,2,3 < 4
L5/S1 level (ii)FF(%)	22.88 ± 7.78	22.87 ± 8.30	24.37 ± 6.54	28.75 ± 9.05	2.631*	1,2,3 < 4
L4/5 level (i) f-CSA	6.54 ± 1.38	6.42 ± 1.10	6.37 ± 1.41	5.51 ± 1.18	2.699*	1 > 2,3,4
L4/5 level (ii) f-CSA	6.50 ± 1.16	6.67 ± 1.39	6.26 ± 1.33	5.43 ± 1.42	3.434*	1 > 2,3,4
L5/S1 level (i) f-CSA	7.30 ± 1.30	7.03 ± 1.27	6.95 ± 1.39	6.10 ± 1.35	2.936*	1 > 2,3,4
L5/S1level (ii) f-CSA	7.52 ± 1.26	7.35 ± 1.45	7.09 ± 1.40	6.32 ± 1.54	2.770*	1 > 2,3,4

Note: (i) means: the affected side; (ii) means: the healthy side. *P < 0.05, **P < 0.01; 1 = age 1 group, 2 = age 2 group, 3 = age 3 group, 4 = age 4 group

### Multifidus degeneration in relation to the lateral aspect of lumbar disc herniation

(1) In patients with lumbar disc herniation at the L4/5 segment, there were no statistically significant differences in FF and f-CSA at the L4/5 and L5/S1 levels of the affected side versus the healthy side (P > 0.05). (2) There were no significant differences in FF at the L4/5 level or f-CSA and FF at the L5/S1 level (P > 0.05) when comparing the affected side with the healthy side in patients with lumbar disc herniation at the L5/S1 segment, whereas the f-CSA at the L5/S1 level was significantly greater on the healthy side than on the affected side (P < 0.05)(Table 5).

**Table 5:** Relationship between degeneration of the multifidus muscle and the lateral aspect of lumbar disc herniation

		Section L4/5						Section L5/S1					
	L4/5 level			L5/S1 level			L4/5 level			L5/S1 level			
	(i)	(ii)	P	(i)	(ii)	P	(i)	(ii)	P	(i)		(ii)	P
FF(%)	22.20 ± 8.54	20.23 ± 7.21	0.0885	23.45 (14.55)	24.57 ± 7.78	0.207	20.07 ± 7.48	20.43 (9.07)	0.117	26.24 ± 9.00		24.69 ± 7.93	0.088
f-CSA	6.26 ± 1.27	6.19 ± 1.13	0.819	6.91 ± 1.23	6.98 ± 1.16	0.422	6.23 ± 1.38	6.24 ± 1.50	0.945	6.84 ± 1.46		7.11 ± 1.59	0.018

Note: (i) means: the affected side; (ii) means: the healthy side

## Discussion

### Relationship between the multifidus and lumbar spine stability

The lumbosacral multifidus is the largest of the paravertebral muscles in terms of area of attachment, namely, in the grooves on either side of the lumbar vertebrae, dorsally attached to the fascia of the lumbar back and extending continuously from the lumbar to the sacrococcygeal region, with a very wide area of action. Morphologically, the multifidus is short, but the muscle belly has a large CSA and is rich in muscle fibres at autopsy [7]. In human studies, the ratio of type I and type II muscle fibres is higher in the multifidus than in the lower limbs, and the higher number of type I muscle fibres provides the basis for the endurance of the multifidus muscle, which allows it to be strong enough to maintain spinal stability [8, 9]. Positionally, the deep muscles of the multifidus are segmentally distributed and are in close contact with the small joints of the lumbar spine in terms of position, thus ensuring that a small moment of force in the multifidus can exert a strong contraction force and that passive tension can stabilise the rotational movements of the lumbar spine, control the smooth progress of intervertebral motion and improve spinal stability [10, 11]. Wilke et al. [12] found that when relative displacement occurs between vertebrae, 2/3 of the spinal stiffness is maintained by the multifidus muscles, with the deep multifidus predominating. In contrast to gross muscles such as the erector spinae and iliopsoas muscles, the multifidus muscle does not focus on the direct movement of the lumbar vertebrae but rather on the contraction of the muscle to counteract undue stresses of spinal rotation or sliding. When the spine is out of balance, the multifidus muscle is the first to contract, increasing tension in the lumbar segments and reducing undesirable displacement and rotation of the spinal segments. The multifidus muscle is therefore closely related to spinal stability.

### Multifidus degeneration varies by sex and age

With age, the multifidus muscle gradually degenerates, showing fatty infiltration, decreased muscle strength and endurance, increased fatigue susceptibility, and a negative correlation with age [13, 14]. Kjaer et al. [15] and Lee et al. [16] found that fatty infiltration of the multifidus muscle was strongly associated with chronic low back pain in adults, whereas in adolescents, the degree of multifidus lipoatrophy was lower and did not correlate significantly with low back pain. Faur Cosmin et al. also found [17] that the CSA of the lumbar multifidus muscle was more atrophic at the L5-S1 level in the healthy population than in the young group. The results of Shahidi et al. [18] suggest that the CSA of the multifidus muscle does not change significantly with age, but its fatty infiltration rate increases significantly, and this change is more pronounced in women. This is supported by the study of Kjaer et al. [15], where the CSA of the multifidus muscle was greater in men than in women, but the degree of fatty infiltration was significantly greater in women than in men.

Our study also found that of the 104 patients in this study, 62 were men and 42 were women. When comparing the fatty ratio of the multifidus muscle in the two groups by sex, the fatty ratio of the multifidus muscle was statistically greater in women than in men, whether on the affected or healthy side of the L4-5 segment or on the affected or healthy side of the L5-S1 segment. This suggests that the degree of fatty infiltration of lumbar disc herniation is significantly higher in women than in men in terms of the degree of lipoatrophy of the multifidus muscle. In terms of age, the lipoatrophy of the multifidus muscle showed an incremental change with age, with a significant increase in lipoatrophy in patients over 50 years of age; the functional CSA of the multifidus muscle showed a negative relationship with age, with the functional CSA of the multifidus muscle shrinking more and more with increasing age, and the functional area of the multifidus muscle shrinking gradually and significantly after the age group of 20–29 years.

### Association of multifidus degeneration with lumbar spine degeneration and lumbar disorders

Degeneration of the multifidus muscle leads to a consequent decrease in lumbar stability and the onset and development of lumbar degeneration and disease.

Kim [19] et al. found that by measuring the CSA of the multifidus muscle in protruding segments of patients with lumbar herniation presenting with significant low back pain, there was a significant reduction in the CSA of the multifidus muscle after 3 months of persistent symptoms of low back pain. Teichtahl AJ et al. [20] found that there was a significant difference between the degree of disc degeneration and the degree of lipid infiltration of the multifidus and erector spinae muscles. In a study by Hu Zexan et al. [21], it was found that in patients with lumbar disc herniation, the degree of fatty infiltration of the affected multifidus muscle increased and the CSA of the multifidus muscle decreased compared to healthy individuals and the healthy side. By observing patients with nonspecific chronic low back pain before and after, Wu Weiwei et al. [22] showed a significant correlation between chronic low back pain and degeneration of the paravertebral multifidus muscle, with a positive correlation between the duration of low back pain, degree of dysfunction and pain and multifidus muscle atrophy. Previous studies have also shown [23] that there is a strong correlation between the degree of degeneration of the multifidus and the site and extent of lumbar spondylolisthesis. The multifidus is also an important factor in maintaining the sagittal balance of the lumbar spine, which is an important factor in maintaining the physiological anterior convexity of the lumbar spine, and the decrease in muscle strength and endurance due to multifidus degeneration affects the curvature of the lumbar spine [24]. Wang et al. [25] found that an imbalance in the sagittal plane of the lumbar spine and a reduction in the anterior convexity of the spine are important factors in inducing a ruptured disc herniation; increased spinal curvature of the lumbar segment is a sensitive factor in inducing degenerative slippage, while the multifidus is an important factor in stabilising the lumbar curvature, and the two are interrelated. Zhao et al. [26], a Japanese scholar, observed 19 patients with unilateral symptomatic lumbar disc herniation and stained sections of the affected and healthy sides of the multifidus muscle and found that both type I and type II fibres on the affected side were significantly smaller than those on the healthy side, and some pathological changes, such as worm-like appearance, were more pronounced on the affected side, where degeneration was more severe. In the present study, there was no statistically significant difference in the lipid ratio or functional CSA at the L4/5 and L5/S1 levels between the affected side and the healthy side of patients with lumbar disc herniation at the L4/5 segment. However, the functional CSA on the healthy side in patients with L5/S1 lumbar disc herniation was significantly greater than that on the affected side. This suggests that the effective use area of the multifidus muscle is stronger on the healthy side than on the affected side in our patients with L5/S1 disc herniation.

The sample size of this case‒control study was small, and the change in multifidus at the L1/2-L3/4 level in patients with L4/5 and L5/S1 disc herniation is unknown; the healthy population was not monitored for multifidus in this study, and a comparison of changes in the multifidus in patients with lumbar disc herniation versus a healthy population is lacking. Although a strict protocol for measuring the fat percentage was followed, there may have been inconsistencies in the observations, leading to biased results. In our next study, we propose to include observations of the paravertebral muscles in a healthy population and the L1/2-L3/4 segment to analyse the relationship between lumbar disc herniation and paravertebral muscles and to provide a basis for optimising treatment options. The lumbar multifidus muscle plays an important role in lumbar spine stability and an irreplaceable role in the pathogenesis and regression of lumbar disc herniation. Therefore, morphological and functional assessment of the multifidus muscle should be emphasised in clinical nonsurgical treatment protocols for lumbar disc herniation, and targeted intervention protocols should be adopted to improve the quality of the multifidus muscle, facilitate the regression of lumbar disc herniation and improve treatment outcomes.

## Data Availability

The datasets generated and/or analysed during the current study are publicly available from the Integrated Platform for Clinical Research (IPCR) of Sichuan Province Orthopedic Hospital and are available from the corresponding author.

## List of abbreviations

LDH: Lumbar disc herniation
FF: fat fraction = fat CSA/total CSA
CSA: The cross-sectional area of the multifidus muscle
f-CSA: functional CSA (defatted CSA of the multifidus muscle)
